# Efficacy of acceptance and commitment therapy (ACT) on depression, pain acceptance, and psychological flexibility in married women with breast cancer: a pre- and post-test clinical trial

**DOI:** 10.47626/2237-6089-2020-0022

**Published:** 2021-01-22

**Authors:** Vajiheh Ghorbani, Zahra Zanjani, Abdollah Omidi, Mostafa Sarvizadeh

**Affiliations:** 1 Kashan University of Medical Sciences Faculty of Medicine Department of Clinical Psychology Kashan Iran Department of Clinical Psychology, Faculty of Medicine, Kashan University of Medical Sciences, Kashan, Iran.; 2 Kashan University of Medical Sciences Department of Medical Oncology Kashan Iran Department of Medical Oncology, Kashan University of Medical Sciences, Kashan, Iran.

**Keywords:** Acceptance and commitment therapy, depression, pain acceptance, psychological flexibility, breast cancer

## Abstract

**Objective:**

Breast cancer is the most common cancer in women worldwide. Many of these patients suffer from multiple psychological symptoms. The present study aimed to investigate the impact of acceptance and commitment therapy (ACT) on depression, pain acceptance, and psychological flexibility in married women with breast cancer.

**Methods:**

The present study was a pre- and post-test clinical trial with intervention and control groups. The research population consisted of women with breast cancer referred to the Ayatollah Yasrebi and Shahid Beheshti Hospitals in Kashan in 2018. Through a purposive sampling method, 40 women were selected and randomly divided into two groups, namely, intervention (n = 20) and control (n = 20). The applied tools included the Depression, Anxiety and Stress Scale (DASS-21), Chronic Pain Acceptance Questionnaire 8 (CPAQ-8), and Acceptance and Action Questionnaire - II (AAQ-II). Data were analyzed by SPSS 16 using descriptive statistics and analysis of variance (ANOVA).

**Results:**

The results showed that ACT treatment significantly reduced the mean scores of depression compared to the control group (F = 107.72, p < 0.001). The mean scores of pain acceptance (F = 9.58, p < 0.05) and psychological flexibility (F = 10.61, p < 0 .05) significantly increased in comparison with the control group.

**Conclusion:**

ACT can be considered as an effective therapeutic approach to reduce depression and increase pain acceptance and psychological flexibility in women with breast cancer. These changes appear to be due to improved acceptance of thoughts and feelings associated with cancer and increased psychological flexibility, which is the primary goal of ACT treatment.

**Clinical trial registration:**

Iranian Registry of Clinical Trials (IRCT), IRCT20190518043620N1.

## Introduction

Breast cancer is the most commonly occurring cancer in women worldwide and has been reported as the second leading cause of cancer deaths. ^1^ It affects Iranian women a decade earlier when compared to their counterparts in developed countries. ^2^^,^^3^ According to statistics, the risk of breast cancer in women worldwide is 22.26%. ^4^ This disease accounts for about one-third of all cancers in women and is the leading cause of death between the ages of 35 and 45. ^5^ The first line of treatment is surgery, but a combination of trastuzumab, pertuzumab, and a kind of chemotherapy called taxane also are applied.^6^

Despite widespread advances in the biological treatment of breast cancer, some problems based on biopsychosocial aspects of this disease remain unsolved. Patients experiencing significant pain and suffering from worry about the future of family members, fear of death, reduced functionality, likelihood of malformations, financial and social problems, body image disorders, and sexual problems are among the factors that affect the mental health of patients with breast cancer. ^5^^,^^7^ In particular, three crucial factors have been leading to widespread disability: depression, pain acceptance, and psychological flexibility.

Studies suggest that depression is one of the most common problems that cancer patients are faced with. ^7^ Some studies have reported that the likelihood of major depression in patients with breast cancer is two times greater than in the general population. ^8^ A study has shown that the death risk in adults with cancer who have depression is higher than among those without depression. ^9^

One of the characteristics of depressed people is the lack of flexibility in different contexts. Psychological flexibility is defined as the ability to adapt to changing environmental stimuli. Some researchers describe it as the ability of the individuals to fully communicate with the present as a conscious human being and their ability to change or continue behaving in the direction of their values. ^10^^-^^12^ What makes a person psychologically vulnerable to depression and anxiety is mental involvement with memories and probable events. ^13^ Studies have shown that psychological flexibility-based therapies effectively improve the physical and psychological symptoms of depression disorders. ^14^ Also, pain is an essential factor leading to disability and reduced quality of life in these patients. Various research studies have demonstrated that medication and rehabilitation do not eliminate the severity of chronic pain. ^15^^-^^17^

Because pain has a bio-psycho-social dimension and consists of a constellation of affective, cognitive, and neural factors, a combination of evidence-based psychotherapies and medication is required for its healing. ^15^^,^^18^ One novel idea in reducing pain-related disability is by teaching the patients how to live with their pain. Research shows that not all patients with chronic pain develop disabilities. Rather, there are people who, despite the pain, continue to perform their usual daily activities. One of the factors contributing to their functional ability is pain acceptance. ^19^ Research has shown that pain acceptance is associated with better social, physical, and psychological functions. ^20^ Therefore, again, combined therapies are necessary in the treatment of breast cancer with the aim of reducing pain-related disability and depression symptoms.

Extensive results have shown that cognitive-behavioral therapy (CBT) is the psychotherapy most commonly used in the medical setting, and it has demonstrated suitable feasibility and efficacy. However, CBT has some limitations. First, it is only concerned with thought contents, whereas clinical backgrounds suggest that most of the patients affected by mental dysfunctions such as depression cannot recognize their cognitive distortion. Second, acceptance is not a CBT component. CBT tries to reduce pain, but sometimes it cannot be reduced and must be accepted. Finally, CBT protocols are applied as symptom-based packages, i.e., they cannot target different comorbid symptoms in one protocol. ^21^^,^^22^ Moreover, empirical evidence retrieved from meta-analyses demonstrated that psychological treatments (such as CBT) in cancer patients (especially breast cancer) generate very small to medium effects on mental health problems. ^23^^-^^25^ Also, another meta-analysis failed to recommend the use of CBT to improve psychological distress and quality of life in breast cancer patients. ^26^ Finally, some studies have shown that CBT is not effective in patients with cancer who experience serious mental health conditions, and that it is more efficient in unmarried participants. ^27^^,^^28^ Based on these limitations, recent studies are exploring the efficacy of a new generation of CBT-based protocols. Acceptance and commitment therapy (ACT) is one on them.

Research has shown that ACT could overcome those obstacles. As a trans-diagnostic approach, ACT deals with the essential pathological core of mental problems rather than specifically with one particular symptom. The ACT therapeutic approach consists of six central processes that lead to psychological flexibility. The six processes include: acceptance, cognitive diffusion, contact with the present moment, self-as-context, values, and committed action. ACT takes its name from its original message, i.e., acceptance of what is out of your control and commitment to taking action that enriches your life. ^21^^,^^29^ Previous studies have demonstrated efficacy of ACT in improving depression and stress in Iranian patients with breast cancer, ^30^^,^^31^ in reducing pain in American patients with breast cancer, ^29^ and in improving both depression and anxiety in breast cancer patients in Colombia. ^32^ However, those studies have some important limitations, such as lack of follow-up period, ^30^^,^^31^ non-random selection, no control for demographic variables, ^31^ small sample size, ^32^ and lack of integrated protocol. ^29^ Also, based on our literature search, there is not any randomized clinical trial currently available targeting these symptoms.

In an attempt to overcome those limitations, the objective of the present study was to determine whether ACT is effective in reducing depression, increasing pain acceptance, and improving psychological flexibility in a sample of Iranian women with breast cancer.

## Methods

### Trial design

The present study was a pre- and post-test clinical trial with intervention and control groups. The intervention group received ACT and was then been compared with patients in a waiting list (control group).

### Participants and randomization

The research population consisted of women with breast cancer referred to the Ayatollah Yasrebi and Shahid Beheshti Hospitals in Kashan in 2018. Using a purposive sampling method, 40 women were selected and randomly divided into experimental (n = 20) and control (n = 20) groups. They were motivated to participate in the program and willing to cooperate with the research team. Informed consent forms were obtained before the initial interview.

### Patient selection

The following inclusion criteria were observed in this study: 1) having a diagnosis of breast cancer by a physician; 2) not presenting other serious diseases (chronic obstructive pulmonary disease, pulmonary disease, diabetes etc.); 3) being at least 18 years old; 4) having at least primary school education level; 5) being married; 6) being motivated to participate in the program; 7) having a depression score ≥ 10 according to the Depression, Anxiety and Stress Scale (DASS-21); 8) having an anxiety score ≥ 8 according to DASS-21 test; and 9) having no history of hospitalization in psychiatric section.

The following exclusion criteria were also taken into consideration: 1) participation in any psychiatric intervention during the course of this study; 2) failure to attend more than two sessions; 3) failure to the homework; 4) reporting suicidal thoughts during the study; and 5) unwillingness to continue the research.

### Procedure

Participants were tested at three time points, namely: before and after the intervention and 2 months after the intervention. In all three assessments, participants were administered the DASS-21, the Chronic Pain Acceptance Questionnaire 8 (CPAQ-8), and the Acceptance and Action Questionnaire - II (AAQ-II). Three external mental health staff members (all with a psychology degree) evaluated the patients’ measures at the three stages (pretest, posttest, and follow-up). They were blinded to the study aims. The intervention was conducted from January 21 2019 to March 18 2019. The follow-up phase started on March 19 2019 and ended on May 8 2020 (2-month follow-up).

### Intervention: ACT

ACT sessions were conducted by a trained psychotherapist with three years of training, with the help of one co-therapist. Both therapists were not aware of the existence of a control group. The protocol of the treatment sessions was based on the book by Hayes et al. ^33^ It consisted of eight weekly sessions lasting 90 minutes each. ^34^ A summary of the contents of each session is presented in Table 1 .

**Table 1:** ACT intervention: contents per session

**First session**	Introducing the members to the therapist and each other, describing group rules, familiarizing with and describing the treatment approach in general.
	**Homework:** Listing 5 examples of the most important problems that patients are faced with in their lives.
**Second session**	Assessment of prior session assignment, assessment of patients’ problems from ACT perspective, extraction of avoidance experience, mixing and individual values.
	**Homework:** Providing a list of the advantages and disadvantages and approaches to problem control.
**Third session**	Assessment of prior session assignment, highlighting the inefficiency of controlling negative events using metaphors and training the tendency toward negative emotions and experiences.
	**Homework:** Registering situations in which the patients could eliminate ineffective control approaches.
**Fourth session**	Assessment of prior session assignment, training distinction between evaluations and personal experiences (bad cup metaphor) and adopting a position of observing the thoughts without judgment.
	**Homework:** Registering situations in which the patients could observe but did not evaluate experiences and emotions.
**Fifth session**	Assessment of prior session assignment, communicating with the present moment and considering it as the context (chessboard metaphor) and teaching mindfulness exercises.
	**Homework:** Registering situations in which the patients were able to observe thoughts using mindfulness techniques.
**Sixth session**	Assessment of prior session assignment, identifying patients’ life values and evaluating values based on their importance.
	**Homework:** Providing a list of obstacles to the realization of values.
**Seventh session**	Assessment of prior session assignment, presenting practical solutions to eliminate the obstacles while applying metaphors and planning for commitment to pursue values.
	**Homework:** Preparing a report on the steps to pursue values and contemplating the outcomes of the sessions.
**Eighth session**	Summing up the concepts discussed during sessions, asking members to explain their achievements to the group and talking about their plans for their lives.

ACT = acceptance and commitment therapy.

### Control group

The control group did not receive any intervention. Instead, the principal investigator informed patients that they would receive therapy 4 months later. After completion of the study, in according with ethical guidelines in research methods, all patients assigned to the control group received ACT in same format and number of sessions.

### Measures

#### Depression, Anxiety, and Stress Scale (DASS-21)

This scale was developed by Lovibond & Lovibond in 1995 and comprises two forms. The short form, used in the present study, contains 21 items, each of which evaluates the psychological constructs of depression, anxiety, and stress, using 7 statements. The final score of each of the subscales is calculated through the sum of the scores of the related items. In one study, the following Cronbach’s alpha values were reported: 0.95 for depression, 0.90 for anxiety, 0.93 for stress, and 0.97 for total score. ^35^ In another study, internal consistency was also assessed using Cronbach’s alpha values, with the following results for depression, anxiety, and stress: 0.77, 0.79, and 0.78, respectively. In criterion validity assessment, the correlation found between DASS-21 and the Beck Depression Inventory was 0.70, with the Zung Self-Rating Anxiety Scale it was 0.67, and with the Perceived Stress Test 0.49. All of these correlations were significant at p < 0.001 level. ^36^

#### Chronic Pain Acceptance Questionnaire (CPAQ)

The Persian version of this tool, like its original version, assesses chronic pain acceptance in two subscales focusing on committed action (11 items) and reluctance to accept pain (9 items). In this scale, scores vary between 0 and 120, and higher scores indicate higher pain acceptance. The instrument has good internal consistency, with a Cronbach’s alpha of 0.82 for committed action and 0.78 for pain acceptance. Moreover, a Cronbach’s alpha coefficient of 0.89 and a test-retest coefficient of 0.71 were reported in the evaluation of the psychometric properties of the Persian version of CPAQ. ^37^^,^^38^

#### Acceptance and Action Questionnaire - II (AAQ-II)

This questionnaire was developed by Bond et al. ^39^ It is a 10-item version of the original questionnaire (AAQ-I) developed by Hayes. This questionnaire measures a construct related to diversity, acceptance, experiential avoidance, and psychological non-flexibility; higher scores indicate higher psychological flexibility. In the psychometric analysis of the original version, results indicated that the instrument had satisfactory reliability, validity, and construct validity. Mean alpha coefficient was 0.84 (0.78 to 0.88) and test-retest reliability was 0.81 and 0.79 in a 3-12-month interval. The tool also shows good discriminant validity. The AAQ-II appears to measure the concept similarly to AAQ-I, but has better psychometric stability. ^40^^,^^41^ Results showed that this scale has suitable internal consistency for use in Iranian populations (0.71 to 0.89). ^42^

### Statistical analysis

Statistical assessment of the demographic information (mean, frequency, and standard deviation) and repeated measures analysis of variance (ANOVA) were conducted using the Statistical Package for the Social Sciences (SPSS) version 16. Significance was set at p = 0.05, and the outcomes were evaluated with a 95% probability.

### Ethical considerations

Before starting the study, a meeting was held with all participants, in separate groups, to present the aims and justification for the study. In this session, ethical matters and information about the study were given to the participants. Then, a consent form was distributed to all patients, which informed that the outcomes and names of the participants would be kept anonymous, and that under no conditions would their medical information be disclosed.

The research protocol for the present study was previously evaluated and approved by the research ethics committee of Kashan University of Medical Sciences (code IR.KAUMS.MEDNT.REC.1397.092). It was also registered in the Iranian Registry of Clinical Trials (IRCT) with code IRCT20190518043620N1.

## Results

In this study, 40 women were randomly assigned to two different groups; there were no dropouts at any of the study stages. Table 2 shows the mean, standard deviation, and frequency percentage of the demographic information collected. The results of the *t* -test showed that there were no significant differences in mean age, length of marriage, or number of children when comparing women with breast cancer in the intervention vs. in the control group. Moreover, education level and occupation were compared using the chi-square test, and no significant differences were found between the two groups for these variables either.

**Table 2:** Demographic characteristics of the study groups

Variables		Control group (n = 20)	Intervention group (n = 20)	p-value
Age, mean (SD)		44.55 (9.12)	46.10 (6.38)	0.53 *
Length of marriage, mean (SD)		22.40 (9.62)	27 (8.01)	0.10 *
Number of children, mean (SD)		2/10 (0.91)	2/40 (1.23)	0.38 *
Education level		2	3	0.61 †
	Primary education			
	Secondary education	7	9	
	University degree	10	6	
	Master	1	2	
	PhD	0	0	
Occupation				
	Employed	3	5	0.42 †
	Housewife	17	15	

SD = standard deviation.

* Independent *t* -test.

† Chi-square test.

The mean and standard deviation values found for depression, pain acceptance, and psychological flexibility at the three evaluation stages are shown in Table 3 . Based on these results, the mean depression score among married women with breast cancer in the ACT group decreased in the post-test compared to the pre-test, and the mean pain acceptance and psychological flexibility scores increased. In addition, the mean depression score in the ACT group increased in the follow-up stage compared to post-test values, but the mean scores of pain acceptance and psychological flexibility further decreased in the follow-up stage compared to the post-test. In the control group, the mean scores of depression increased, whereas the mean scores of pain acceptance and psychological flexibility decreased, in the post-test compared to the pre-test and also in the follow-up compared to the post-test.

**Table 3:** Mean and standard deviation values for depression, pain acceptance and psychological flexibility in the two study groups at different stages of evaluation

Variables	Control group			Intervention group		
	Pre-test	Post-test	Follow-up	Pre-test	Post-test	Follow-up
Depression	27.70 (±6.56)	29.10 (±4.07 )	31.70 (±3.38)	29.10 (±7.29)	11.90 (±3.40)	13.20 (±3.33)
Pain acceptance	53.05 (±6.86)	55.35 (±6.51)	57.55 (±6.72)	52 (±7.80)	68.70 (±15.89)	71.70 (±17.54)
Psychological flexibility	26.10 (±8.8 )	27.50 (±9.64)	29.45 (±9.57)	28.25 (±11.28)	39.40 (±11.57)	42.15 (±9.30)

The use of parametric tests, such as analysis of covariance (ANCOVA), requires observing a set of presumptions. In this study, the subjects were randomly assigned to the two groups. Due to use of standard tools for evaluating the dependent variables, the presupposition of the interval nature of the dependent variable was observed and also the normality of the scores distribution through the Kolmogorov-Smirnov test, the equality of the variance scores in the pre-test by Levine test, and the equality of the covariance of the variables in the pre-test were followed by the M Box test. Table 4 shows the results of repeated measures ANOVA. As it seen in this table, there were significant differences in severity of depression symptoms (F = 107.72, p < 0.001), mean changes of pain acceptance (F = 9.58, p < 0.05) and mean changes of psychological flexibility (F = 10.61, p < 0.05) in the intervention group.

**Table 4:** Results of the analysis of variance with repeated measurement of depression scores, pain acceptance and psychological flexibility

Variables	Time			Group			Time × group		
	F	p-value	Eta effect size	F	p-value	Eta effect size	F	p-value	Eta effect size
Depression	36.47	p < 0.001	0.49	107.72	p < 0.001	0.73	66.37	p < 0.001	0.63
Pain acceptance	24.06	p < 0.001	0.38	9.58	0.004	0.20	10.85	p < 0.001	0.22
Psychological flexibility	19.67	p < 0.001	0.34	10.61	0.002	0.21	8.53	0.005	0.18

## Discussion

The current study aimed to investigate the effect of ACT on depression, pain acceptance, and psychological flexibility in married women with breast cancer. Analysis of results indicated that this treatment could reduce depression and increase pain acceptance and psychological flexibility in these patients. The results are in line with the findings of many studies on the impact of ACT. For instance, Mojtabaie & Gholamhosseini assessed women with breast cancer and depressive symptoms, and found that the treatment effectively reduced symptoms of depression in the patients. ^43^ Findings from ACT in the study of Mohabbat-Bahar et al. suggested a significant reduction in anxiety and depression symptoms in women with breast cancer. ^31^ In a study by Mani et al. involving women with breast cancer, the authors found that ACT effectively reduced the severity of perceived pain and increased pain acceptance. ^44^ Finally, the results of ACT in the study by Najvani et al. indicated a significant decrease in symptoms of depression and increased psychological flexibility in these women. ^34^ Although ACT does not directly reduce symptoms (for example, depression symptoms), it is believed that if patients do not try to decrease their thoughts and feelings, and instead try to abandon the struggle with them and move on with their lives’ values, then symptoms will decrease spontaneously. ^45^

According to Hayes et al., more than half of the changes in depressive symptoms can be explained by non-acceptance and desire. ^45^ Concerning the effectiveness of the ACT in reducing pain in patients with chronic pain, it can be suggested that, in people with breast cancer, the way how people interpret or respond to their pain is crucial in the experience of pain. Catastrophizing pain creates an exaggerated and negative tendency toward real pain and directs people to spend their lives without it. This phenomenon is known as experiential avoidance in ACT. However, research shows that this approach to pain makes it more valuable, more dominant, and more annoying, which is the same definition as pain turning to suffering; what happens subsequently is the loss of life and its meaning. Conversely, according to Batten et al., the success of ACT in treating different clinical types and different groups of people is that this approach focuses more on the functional processes behind the disrupted behaviors rather than on the characteristic symptoms of a disorder. ^46^ This treatment examines behavioral patterns that hinder success in life. The therapist focuses more on the overall improvement of the patient’s life, rather than on reducing symptoms. ACT aims to improve behavior in the presence of frightening internal events (e.g., distressing thoughts and feelings related to cancer), something called psychological flexibility. ^33^

One fundamental process in ACT is clarifying the values of each individual. This process is defined as intrinsic reinforcement, meaning that, despite the obstacles, the subject’s values provide a choice for one’s behaviors and actions. Because cancer patients face life-threatening diseases, they may often become unaware of their values. Therefore, helping individuals to detect their values and precious areas in life provides a motive to move on and seriously deal with cancer diagnosis and treatment. This, in turn, can lead to better medical and psychological outcomes. By clarifying values and goals, patients will realize that for years, instead of trying things that can change them (i.e., functioning according to their values), they have tried unchangeable things (i.e., thoughts, emotions, memories of cancer, etc.). Through ACT patients are helped to accept, embrace, and engage in behavior that incorporates their values, rather than fighting their thoughts and feelings. Thus, even though the disease may still limit their activities, they will start behaving consistently with their values, e.g., by communicating with others and improving relationships with family, friends, religion, and spirituality. Active involvement in invaluable areas of life leads to improved medical and psychological outcomes. Evidence suggests that increased psychological flexibility also improves the patient’s psychological distress. In other words, it seems that the process of acceptance and mindfulness, and the process of changing behavior, end up improving the morale of patients with breast cancer. ^21^^,^^30^^,^^33^

This study’s limitations are the use of self-report tools and the lack of consideration of other mental and psychological problems in women with breast cancer. Also, because of the difficulty associated with investigating these patients, the sample size was small. One possible way around the small sample size would be to offer ACT-based psychological treatment in specialized cancer treatment units and also at outpatient treatment clinics that see both men and women in different age groups. Assessing larger groups is suggested to validate the approach here presented. Finally, it would be interesting to compare this approach with other therapies and to evaluate their therapeutic effect over a more extended follow-up period.

## Conclusion

According to the results, ACT was an effective therapeutic approach to reduce depression and increase pain acceptance and psychological flexibility in women with breast cancer. These changes appear to be achieved through increased acceptance of thoughts and feelings associated with cancer and increased psychological flexibility, which is the primary goal of ACT treatment.
